# Efficient genome-scale phylogenetic analysis under the duplication-loss and deep coalescence cost models

**DOI:** 10.1186/1471-2105-11-S1-S42

**Published:** 2010-01-18

**Authors:** Mukul S Bansal, J Gordon Burleigh, Oliver Eulenstein

**Affiliations:** 1 School of Computer Science, Tel Aviv University, Tel Aviv 69978, Israel; 2 Department of Biology, University of Florida, Gainesville, FL 32611, USA; 3 Department of Computer Science, Iowa State University, Ames, IA 50011, USA

## Abstract

**Background:**

Genomic data provide a wealth of new information for phylogenetic analysis. Yet making use of this data requires phylogenetic methods that can efficiently analyze extremely large data sets and account for processes of gene evolution, such as gene duplication and loss, incomplete lineage sorting (deep coalescence), or horizontal gene transfer, that cause incongruence among gene trees. One such approach is gene tree parsimony, which, given a set of gene trees, seeks a species tree that requires the smallest number of evolutionary events to explain the incongruence of the gene trees. However, the only existing algorithms for gene tree parsimony under the duplication-loss or deep coalescence reconciliation cost are prohibitively slow for large datasets.

**Results:**

We describe novel algorithms for SPR and TBR based local search heuristics under the duplication-loss cost, and we show how they can be adapted for the deep coalescence cost. These algorithms improve upon the best existing algorithms for these problems by a factor of *n*, where *n *is the number of species in the collection of gene trees. We implemented our new SPR based local search algorithm for the duplication-loss cost and demonstrate the tremendous improvement in runtime and scalability it provides compared to existing implementations. We also evaluate the performance of our algorithm on three large-scale genomic data sets.

**Conclusion:**

Our new algorithms enable, for the first time, gene tree parsimony analyses of thousands of genes from hundreds of taxa using the duplication-loss and deep coalescence reconciliation costs. Thus, this work expands both the size of data sets and the range of evolutionary models that can be incorporated into genome-scale phylogenetic analyses.

## Background

The availability of large-scale genomic data sets provides an unprecedented wealth of information for phylogenetic analyses. However, genomic data sets also present a number of unique challenges for phylogenetics. Beyond the exceptional computational challenges involved in analyses of thousands of genes from many taxa, complex patterns of gene evolution within the genome, including incomplete lineage sorting (deep coalescence), gene duplications and losses, lateral gene transfer, and recombination, create tremendous heterogeneity in the topology of gene trees and obscure species relationships [1]. In fact, in some evolutionary scenarios, trees from the majority of genes in a genome can differ from the species phylogeny (e.g., [2]). Thus, methods for incorporating genomic data into phylogenetic analyses must be both computationally tractable for extremely large data sets and must account for heterogeneous processes of gene family evolution. In this paper, we introduce novel algorithms that enable for the first time estimates of phylogenetic trees from large genomic data sets based on gene duplications and losses as well as incomplete lineage sorting.

One approach for building phylogenetic trees from genomic data sets is gene tree parsimony (GTP). Given a collection of gene trees, GTP seeks a species tree that implies the minimum reconciliation cost, or number of events that cause conflict among the gene trees. However, available GTP algorithms either lack sufficient speed to be useful for large data sets or the flexibility to deal with a wide range of evolutionary processes that affect gene tree topologies. In particular, the *duplication-loss problem *[3-13], which is the GTP problem based on minimizing the number of gene duplications and losses, and the *deep-coalescence problem *[1,14-16], which is the GTP problem based on minimizing the number of deep coalescences, lack efficient heuristics.

Both the duplication-loss problem and the deep-coalescence problem are NP-hard [15,17]. Therefore, in practice, these problems are typically approached using local search heuristics. These heuristics start with some initial candidate species tree and find a minimum reconciliation cost tree in its neighborhood. This constitutes one local search step. The best tree thus found then becomes the starting point for the next local search step, and so on, until a local minima is reached. Thus, at each local search step, the heuristic solves a "local search problem". The time complexity of this local search problem depends on the tree edit operation used to define the neighborhood.

Rooted subtree pruning and regrafting (SPR) [18] and rooted tree bisection and reconnection (TBR) [19] are two of the most effective and most commonly used tree edit operations. For example, Page [20] and Maddison and Knowles [14] implemented heuristics based on the SPR local tree search to estimate the species tree that minimizes the number of deep coalescences. Similarly, SPR based local search heuristics were also developed for the duplication-loss problem [20,21]. However, these heuristics estimate the reconciliation cost from scratch for each tree topology that is evaluated, and therefore, they are only useful for small data sets. Recently, Than and Nakhleh [16] developed an integer linear programming formulation and a dynamic programming algorithm to provide exact solutions for the deep coalescence problem. Although these exact algorithms can analyze data sets with hundreds of gene trees, they are limited to a very small number of taxa. Furthermore, while there do exist fast heuristics based on SPR [22] and TBR [23] local searches for GTP, these are based on minimizing the gene duplication cost only.

Several methods exist for inferring species trees from collections of conflicting genes in a probabilistic framework, but, these are similarly limited. Liu and Pearl [24] and Kubatko et al. [25] discuss likelihood-based approaches to estimate a species tree from gene trees based on a coalescent process. These methods, along with Ané et al.'s [26] Bayesian approach to estimating concordance among gene trees, require gene trees with a single gene per taxon, and they have only been tested on small data sets. In addition, probabilistic models of gene tree-species tree reconciliation that incorporate duplication and loss also exist [27,28]. However, these models are computationally complex, and they have not been incorporated in any tree search heuristics.

### Our contributions

A lack of efficient heuristics has limited the use of the GTP approach for phylogenetic analyses of large-scale genomic data sets, and there are no options for such GTP analyses based on the duplication-loss and deep-coalescence problems. In this paper, we address this issue by presenting efficient, novel algorithms for SPR and TBR based local searches for both of these problems. Let us assume, for convenience, that the size of the *k *given gene trees differs by a constant factor from the size of the resulting species tree. The currently best known (naïve) solutions for the SPR and TBR local search problems, for the duplication-loss as well as the deep-coalescence problem, require Θ(*kn*^3^) and Θ(*kn*^4^) time respectively, where *n *is the size of the resulting species tree. Our new algorithms solve these SPR and TBR local search problems in *O*(*kn*^2^) and *O*(*kn*^3^) time respectively. Consequently, our algorithms provide a speedup of a factor of *n *over the best known SPR and TBR local search algorithms (like the ones implemented in [20,21]) for the duplication-loss and deep-coalescence problems. This enables, for the first time, GTP analyses with hundreds of taxa and thousands of genes based on gene duplications and losses or incomplete lineage sorting.

We first develop our algorithms in the context of the duplication-loss problem, and then show how to apply them to the deep-coalescence problem. Then, we demonstrate the improvement in runtime and scalability provided by our algorithms over the best current solutions by using an implementation of our algorithm for the SPR local search.

## Methods

### Basic notation and preliminaries

Given a rooted tree *T*, we denote its node set, edge set, and leaf set by *V *(*T*), *E*(*T*), and *Le*(*T*) respectively. The root node of *T *is denoted by *rt*(*T*). Given a node *v *∈ *V *(*T*), we denote its parent by *pa*_*T *_(*v*), its set of children by *Ch*_*T *_(*v*), and the subtree of *T *rooted at *v *by *T*_*v*_. If two nodes in *T *have the same parent, they are called *siblings*. The set of *internal nodes *of *T*, denoted *I*(*T*), is defined to be *V *(*T*)\*Le*(*T*). We define ≤ _*T *_to be the partial order on *V *(*T*) where *x *≤_*T *_*y *if *y *is a node on the path between *rt*(*T*) and *x*. The *least common ancestor *of a non-empty subset *L *⊆ *V *(*T*) in tree *T*, denoted as lca_*T *_(*L*), is the unique smallest upper bound of *L *under ≤_*T*_.

Given *x*, *y *∈ *V *(*T*), *x *→_*T *_*y *denotes the unique path from *x *to *y *in *T*. We denote by *d*_*T *_(*x*, *y*) the number of edges on the path *x *→_*T *_*y*. *T *is fully *binary *if every node has either zero or two children. Throughout this paper, the term tree refers to a rooted fully binary tree. Given *T *and a set *L *⊆ *Le*(*T*), let *T' *be the minimal rooted subtree of *T *with leaf set *L*. We define the leaf induced subtree *T*[*L*] of *T *on leaf set *L *to be the tree obtained from *T' *by successively removing each non-root node of degree two and adjoining its two neighbors.

#### The Duplication-Loss problem

A *species tree *is a tree that depicts the evolutionary relationships of a set of species. Given a gene family for a set of species, a *gene tree *is a tree that depicts the evolutionary relationships among the sequences encoding only that gene family in the given set of species. Thus, the nodes in a gene tree represent genes. We assume that each leaf of the gene trees is labeled with the species from which that gene was sampled. In order to compare a gene tree *G *with a species tree *S*, we require a mapping from each gene *g *∈ *V *(*G*) to the most recent species in *S *that could have contained *g*.

**Definition 1 **(Mapping). *The *leaf-mapping ℒ_*G*, *S *_: *Le*(*G*) → *Le*(*S*) *maps a leaf node g *∈ *Le*(*G*) *to that unique leaf node s *∈ *Le*(*S*) *which has the same label as g. The extension *ℳ_*G*, *S *_: *V *(*G*) → *V *(*S*) *of *ℒ_*G*, *S *_*is the *mapping *defined by *ℳ_*G*, *S*_(*g*) = lca(ℒ_*G*, *S*_(*Le*(*G*_*g*_))).

For any node *s *∈ *V *(*S*), we use  to denote the set of nodes in *G *that map to node *s *∈ *V *(*S*) under the mapping ℳ_*G*, *S*_.

**Definition 2 **(Comparability). *Given trees G and S, we say that G is *comparable *to S if, for each g *∈ *Le*(*G*), *the leaf-mapping *ℒ_*G*, *S*_(*g*) *is well defined. A set of gene trees **is *comparable *to S if each gene tree in **is comparable to S*.

Throughout this paper we use the following terminology:  is a set of gene trees that is comparable to a species tree *S*, and *G *∈ .

**Definition 3 **(Duplication). *A node g *∈ *I*(*G*) *is a *(gene) duplication *if *ℳ_*G*, *S*_(*g*) ∈ ℳ_*G*, *S*_(*Ch*(*g*)) *and we define Dup*(*G*, *S*) = {*g *∈ *I*(*G*): *g is a duplication*}.

Following [8], we define the number of losses as follows.

**Definition 4 **(Losses). *The number of *losses *Loss*(*G*, *S*, *g*) *at a node g *∈ *I*(*G*), *is defined to be:*

• 0, *if *ℳ_*G*, *S' *_(*g*) = ℳ_*G*, *S*″ _(*g'*) ∀ *g' *∈ *Ch*(*g*), *and*

• ∑_*g'*∈*Ch*(*g*)_| *d*_*S' *_(ℳ_*G*, *S' *_(*g*), ℳ_*G*, *S' *_(*g'*)) - 1|, *otherwise;*

*where S' *= *S*[*Le*(*G*)]. *We define Loss*(*G*, *S*) = Σ_*g*∈*I*(*G*) _*Loss*(*G*, *S*, *g*) *to be the number of losses in G*.

Under the duplication-loss model, the reconciliation cost of *G *with *S *is simply the duplication-loss cost; i.e., the number of duplications and losses.

**Definition 5 **(Reconciliation cost). *We define reconciliation costs for gene and species trees as follows:*

*1. *Δ(*G*, *S*) = | *Dup*(*G*, *S*)| + *Loss*(*G*, *S*) *is the *reconciliation cost from *G *to *S*.

*2. *Δ(, *S*) =Σ_*G*∈_Δ(*G*, *S*) *is the *reconciliation cost *from **to S*.

*3. Let **be the set of species trees that are comparable with *. *We define **to be the *reconciliation cost of .

**Problem 1 **(Duplication-Loss). *Given a set **of gene trees, the *Duplication-Loss problem is to find a species tree S* *comparable with *, *such that Δ *(, S*) = Δ().

#### Local search problems

Here we first provide the definition of an SPR edit operation [18] and then formulate the related local search problems. The definition and associated local search problems for the TBR edit operation are considered later.

For technical reasons, before we can define the SPR operation, we need the following definition.

**Definition 6 **(Planted tree). *Given a tree T*, *the *planted tree Φ(*T*) *is the tree obtained by adding a *root edge {*p*, *rt*(*T*)}, *where p *∉ *V *(*T*), *to T*.

**Definition 7 **(SPR operation). *Let T be a tree, e *= {*u*, *v*} ∈ *E*(*T*), *where u *= *pa*(*v*), *and X*, *Y be the connected components that are obtained by removing edge e from T such that v *∈ *X and u *∈ *Y*. *We define *SPR_*T *_(*v*, *y*) *for y *∈ *Y to be the tree that is obtained from Φ*(*T*) *by first removing edge e, and then adjoining a new edge f between v and Y as follows:*

*1. Create a new node y' that subdivides the edge *{*pa*(*y*), *y*}.

*2. Add edge f between nodes v and y'*.

*3. Suppress the node u, and rename y' as u*.

*4. Contract the root edge*.

*We say that the tree *SPR_*T *_(*v*, *y*) *is obtained from T by a *subtree prune and regraft (SPR) *operation that *prunes *subtree T*_*v *_*and *regrafts *it above node y*.

**Notation. **We define the following:

*1. *SPR_*T *_(*v*) =∪_*y*∈*Y*_{SPR_*T *_(*v*, *y*)}

*2. *SPR_*T *_= ∪ _(*u*, *v*)∈*E*(*T*) _SPR_*T *_(*v*)

Throughout the remainder of this manuscript, *S *denotes a species tree such that , and *v *is a non-root node in *V *(*S*).

We now define the relevant local search problems based on the SPR operation.

**Problem 2 **(SPR-Scoring (SPR-S)).

*Given *and S, find a tree T *∈ SPR_*S *_*such that *Δ (, T *) =  Δ (, *T*).

Our goal is to solve the SPR-S problem efficiently. To that end, we first define a restricted version of the SPR-S problem, called the SPR-*Restricted Scoring *problem.

**Problem 3 **(SPR-Restricted Scoring (SPR-RS)).

*Given *, S, and v, find a tree T *∈ SPR_*S*_(*v*) *such that Δ *(, T *) =  Δ(, *T*).

Let *n *= | *Le*(*S*)|, *m *= | *Le*(*S*)| + | *Le*(*G*)| and *k *= ||, and let us assume, for convenience, that all *G *∈  have approximately the same size. In the following, we show how to solve the SPR-RS problem in *O*(*km*) time. Since SPR_*S *_= ∪ _{*pa*(*v*), *v*} ∈ *E*(*S*) _SPR_*S*_(*v*), it is easy to see that the SPR-S problem can be solved by solving the SPR-RS problem *O*(*n*) times. This yields an *O*(*kmn*) time algorithm for the SPR-S problem. Later, we show that the local search problem corresponding to the TBR operation reduces to solving *O*(*n*^2^) SPR-RS problems; which yields an *O*(*kmn*^2^) time algorithm for the TBR local search problem. In the interest of brevity, all lemmas and theorems in this paper appear with proofs omitted; however, all proofs are available in [29].

### Solving the SPR-RS problem

Throughout this section, we limit our attention to one gene tree *G*; in particular, we show how to solve the SPR-RS problem for *G *in *O*(*m*) time. Our algorithm extends trivially to solve the SPR-RS problem on the set of gene trees  in *O*(*km*) time. For simplicity, we will assume that *Le*(*G*) = *Le*(*S*). Indeed, if *Le*(*G*) ≠ *Le*(*S*) then we can simply set the species tree to be *S*[*Le*(*G*)]; this takes *O*(*n*) time and, consequently, does not affect the time complexity of our algorithm.

In order to solve the SPR-RS problem for *G*, it is sufficient to compute the values | *Dup*(*G*, *S'*)| and *Loss*(*G*, *S'*) for each *S' *∈ SPR_*S*_(*v*). Bansal et al. [22] showed how to compute the value | *Dup*(*G*, *S'*)| for each *S' *∈ SPR_*S*_(*v*), in *O*(*m*) time. Losses, however, behave in a very different and much more complex manner compared to gene duplications and it has remained unclear if their computation could be similarly optimized. In this paper we show that it is indeed possible to compute the value *Loss*(*G*, *S'*) for each *S' *∈ SPR_*S*_(*v*) in *O*(*m*) time as well. Altogether, this implies that the SPR-RS problem for *G *can be solved in *O*(*m*) time. Next, we introduce some of the basic structural properties that are helpful in the current setting.

#### Basic structural properties

Consider the tree *N*^*S*, *v *^= SPR_*S*_(*v*, *rt*(*S*)). Observe that, since  = SPR_*S*_(*v*), solving the SPR-RS problem on instance ⟨{*G*}, *S*, *v*⟩ is equivalent to solving it on the instance ⟨{*G*}, *N*^*S*, *v*^, *v*⟩. Thus, in the remainder of this section, we will work with tree *N*^*S*, *v *^instead of tree *S*; the reason for this choice becomes clear in light of Lemmas 3 and 4.

Since *S *and *v *are fixed in the current context, we will, in the interest of clarity, abbreviate *N*^*S*, *v *^simply to *N*. Similarly, in the remainder of this section, we abbreviate ℳ_*G*, *T *_to ℳ_*T*_, for any species tree *T*.

Throughout the remainder of this work, let *u *denote the sibling of *v *in *N*. We color the nodes of *N *asfollows: (i) All nodes in the subtree *N*_*v *_are colored red, (ii) the root node of *N *is colored blue, and (iii) all the remaining nodes, i.e. all nodes in *N*_*u*_, are colored green. Correspondingly, we color the nodes of *G *by assigning to each *g *∈ *V *(*G*) the color of the node ℳ_*N*_(*g*).

**Definition 8 **(Γ). *We define *Γ *to be the tree obtained from G by removing all red nodes (along with any edges incident on these red nodes). Observe that while Γ must be binary, it might not be *fully *binary*.

The significance of the tree Γ stems from the following two Lemmas which, together, completely characterize the mappings from nodes in *V *(*G*) for each *S' *∈ SPR_*S*_(*v*). This characterization is the basis of Lemmas 3 through 8.

**Lemma 1**. *Given G and N, if g *∈ *V *(*G*) *is either red or green, then *ℳ_*S'*_(*g*) = ℳ_*N*_(*g*) *for all S' *∈ SPR_*N *_(*v*).

**Lemma 2**. *Given G and N, if g *∈ *V *(*G*) *is a blue node, then *ℳ_*S' *_(*g*) = lca_*S' *_(*v*, ℳ_Γ, *N *_(*g*)) *for any S' *∈ SPR*N*(*v*).

#### Characterizing losses

To solve the SPR-RS problem efficiently we rely on the following six lemmas, which make it possible to efficiently infer the value of *Loss*(*G*, *S'*, *g*) for any *S' *∈ SPR_*N *_(*v*) and any *g *∈ *V *(*G*).

Consider any *g *∈ *I*(*G*), and let *g' *and *g" *be its two children. Let *a *= ℳ_*N*_(*g*), *b *= ℳ_*N *_(*g'*) and *c *= ℳ_*N *_(*g"*). Without loss of generality, node *g *must correspond to one of the following six categories: 1) *g *is red, 2) *g *is green, 3) *g*, *g'*, and *g" *are all blue, 4) *g *and *g' *are blue, and *g" *is green, 5) *g *and *g' *are blue, and *g" *is red, or, 6) *g *is blue, *g' *is red, and *g" *is green.

Lemmas 3 through 8 characterize the behavior of the loss cost *Loss*(*G*, *S'*, *g*), for each *S' *∈ SPR_*N *_(*v*), for each of these six cases. At this point, it would help to observe that SPR_*N *_(*v*) = {SPR_*N *_(*v*, *s*): *s *∈ *V *(*N*_*u*_)}.

**Lemma 3**. *If g is red then Loss*(*G*, *S'*, *g*) = *Loss*(*G*, *N*, *g*) *for all S' *∈ SPR_*N *_(*v*).

**Lemma 4**. *If g is green then Loss*(*G*, *S'*, *g*) = *Loss*(*G*, *N*, *g*) + 1 *if S' *= SPR_*N *_(*v*, *x*) *where b *≤_*N *_*x *<_*N *_*a or c *≤_*N *_*x *<_*N *_*a, and Loss*(*G*, *S'*, *g*) = *Loss*(*G*, *N*, *g*) *otherwise*.

**Lemma 5**. *Let g, g' and g" all be blue nodes, x *∈ *V *(*N*_*u*_), *and let a' *= ℳ_Γ, *N *_(*g*), *b' *= ℳ_Γ, *N *_(*g'*) *and c' *= ℳ_Γ, *N *_(*g"*).

*1. If S' *= SPR_*N *_(*v*, *x*) *where x *≮_*N *_*a', then Loss*(*G*, *S'*, *g*) = *Loss*(*G*, *N*, *g*).

*2. If S' *= SPR_*N *_(*v*, *x*) *where x *<_*N *_*a', and S" *= SPR_*N *_(*v*, *pa*(*x*)), *then*,

*(a) Loss*(*G*, *S'*, *g*) = *Loss*(*G*, *S"*, *g*) + 1 *if b' *≤_*N *_*x *<_*N *_*a' or c' *≤_*N *_*x *<_*N *_*a', and*,

*(b) Loss*(*G*, *S'*, *g*) = *Loss*(*G*, *S"*, *g*) *otherwise*.

**Lemma 6. ***Let g and g' be blue nodes and g" be a green node, x *∈ *V *(*N*_*u*_)\{*u*}, *and let a' *= ℳ_Γ, *N *_(*g*), *b' *= ℳ_Γ, *N *_(*g'*) *and c' *= ℳ_Γ, *N *_(*g"*).

*1. If S' *= SPR_*N *_(*v*, *x*) *where x *≮_*N *_*a', and S" *= SPR_*N *_(*v*, *pa*(*x*)), *then*,

*(a) Loss*(*G*, *S'*, *g*) = *Loss*(*G*, *S"*, *g*) - 1 *if a' *≤_*N *_*x *<_*N *_*u*,

*(b) Loss*(*G*, *S'*, *g*) = *Loss*(*G*, *S"*, *g*) - 1 *if a' *≤ _*N *_*pa*(*x*) <_*N *_*u but x is not such that a' *≤_*N *_*x *<_*N *_*u*, *and*,

*(c) Loss*(*G*, *S'*, *g*) = *Loss*(*G*, *S"*, *g*) *otherwise*.

*2. Let S' *= SPR_*N *_(*v*, *x*) *where x *<_*N *_*a' and S" *= SPR_*N *_(*v*, *pa*(*x*)).

*(a) If a' *≠ *b' and b" denotes the child of a' along the path a' *→ _*N *_*b'*, *then*,

*i. Loss*(*G*, SPR_*N *_(*v*, *b"*), *g*) = *Loss*(*G*, SPR_*N *_(*v*, *a'*), *g*) - 2 *if a' *≠ *c'. And*,

   *Loss*(*G*, SPR_*N *_(*v*, *b"*), *g*) = *Loss*(*G*, SPR_*N *_(*v*, *a'*), *g*) *if a' *= *c'*,

*ii. Loss*(*G*, *S'*, *g*) = *Loss*(*G*, *S"*, *g*) + 1 *if b' *≤ _*N *_*x *<_*N *_*b"*,

*iii. Loss*(*G*, *S'*, *g*) = *Loss*(*G*, *S"*, *g*) *if x is such that x *∈ *V *(*N*_*b*"_) *but not such that b' *≤ _*N *_*x *<_*N *_*a'*,

*iv. Loss*(*G*, *S'*, *g*) = *Loss*(*G*, SPR_*N *_(*v*, *a'*), *g*) *if c' *≤_*N *_*x *<_*N *_*a'*, *and*,

*v. Loss*(*G*, *S'*, *g*) = *Loss*(*G*, SPR_*N *_(*v*, *a'*), *g*) -1 *otherwise*.

*(b) If a' *= *b'*, *then*,

*i. Loss*(*G*, *S'*, *g*) = *Loss*(*G*, SPR_*N *_(*v*, *a'*), *g*) *if c' *≤ _*N *_*x *<_*N *_*a'*, *and*,

*ii. Loss*(*G*, *S'*, *g*) = *Loss*(*G*, SPR_*N *_(*v*, *a'*), *g*) -1 *otherwise*.

**Lemma 7**. *Let g and g' be blue nodes and c be a red node, x *∈ *V *(*N*_*u*_), *and let a' *= ℳ_Γ, *N*_(*g*).

*1. If S' *= SPR_*N *_(*v*, *x*) *where x *<_*N *_*a'*, *and S" *= SPR_*N *_(*v*, *pa*(*x*)), *then Loss*(*G*, *S'*, *g*) = *Loss*(*G*, *S"*, *g*) + 1.

*2. If S' *= SPR_*N *_(*v*, *x*) *where x *≮_*N *_*a'*, *then*,

*(a) Loss*(*G*, *S'*, *g*) = *Loss*(*G*, *N*, *g*) *if a' *≤_*N *_*x *≤_*N *_*u, and*,

*(b) Loss*(*G*, *S'*, *g*) = *Loss*(*G*, *S"*, *g*) + 1 *for S" *= SPR_*N *_(*v*, *pa*(*x*)) *otherwise*.

**Lemma 8. ***Let g be blue, g' be red, and g'' be green. Let x *∈ *V *(*N*_*u*_)\{*u*} *and a' *= ℳ_Γ, *N *_(*g*).

*1. If S' *= SPR_*N *_(*v*, *x*) *where x *≮_*N *_*a'*, *and S" *= SPR_*N *_(*v*, *pa*(*x*)), *then*,

*(a) Loss*(*G*, *S'*, *g*) = *Loss*(*G*, *S"*, *g*) - 1 *if a' *≤ _*N *_*x *<_*N *_*u*,

*(b) Loss*(*G*, *S'*, *g*) = *Loss*(*G*, *S"*, *g*) *if a' *≤ _*N *_*pa*(*x*) <_*N *_*u but x is not such that a' *≤_*N *_*x *<_*N *_*u, and*,

*(c) Loss*(*G*, *S'*, *g*) = *Loss*(*G*, *S"*, *g*) + 1 *otherwise*.

*2. If S' *= SPR_*N *_(*v*, *x*) *where x *<_*N *_*a'*, *and S" *= SPR_*N *_(*v*, *pa*(*x*)), *then*,

*(a) Loss*(*G*, *S'*, *g*) = *Loss*(*G*, SPR_*N *_(*v*, *a'*), *g*) + 2 *if x *∈ *Ch*_*N *_(*a'*), *and*,

*(b) Loss*(*G*, *S'*, *g*) = *Loss*(*G*, *S"*, *g*) + 1 *otherwise*.

### The algorithm

Observe that SPR_*N *_(*v*) = {SPR_*N *_(*v*, *s*): *s *∈ *V *(*N*_*u*_)}. Therefore, the goal of our algorithm is to compute at each node *s *∈ *V *(*N*_*u*_) the value *Loss*(*G*, *S'*), where *S' *= SPR_*N *_(*v*, *s*). To do this efficiently, we rely on the characterization of losses given in Lemmas 3 through 8.

The first step of the algorithm is to compute the value *Loss*(*G*, *N*). This "loss value" is assigned to the node *u*. To compute the loss value for the rest of the nodes our algorithm makes use of six different types of *counters *at each node in *N*_*u*_; we refer to these counters as counter-*i*, for *i *∈ {1,..., 6}. The reason for using these six counters is that the behavior of the loss values can be explained by using six types of patterns (captured by the six counters). These counters make it possible to efficiently compute the difference between the values *Loss*(*G*, *N*) and *Loss*(*G*, *S'*), where *S' *= SPR_*N *_(*v*, *s*), for each *s *∈ *V *(*N*_*u*_). Next, we describe each of these six counters; throughout our description, *s *represents some node in *N*_*u*_.

**counter-1 **If the value of counter-1 is *x *at node *s *then this implies that the tree SPR_*N *_(*v*, *s*) incurs *x *additional losses over the value *Loss*(*G*, *N*).

**counter-2 **If the value of counter-2 is *x *at node *s*, then this implies that for each *t *≤_*N *_*s *the tree SPR_*N *_(*v*, *t*) incurs an additional *x *losses over *Loss*(*G*, *N*).

**counter-3 **If the value of counter-3 is *x *at node *s*, then this implies that for each *t≤ *_*N *_*s *the tree SPR_*N *_(*v*, *t*) loses *x *losses from *Loss*(*G*, *N*).

**counter-4 **If the value of counter-4 is *x *at node *s*, then this implies that for each *t *≤ _*N *_*s *the tree SPR_*N *_(*v*, *t*) incurs *α*_*t *_· *x *additional losses over *Loss*(*G*, *N*, where *α*_*t *_= *d*_*N *_(*pa*(*s*), *t*).

**counter-5 **If the value of counter-5 is *x *at node *s*, then it is equivalent to incrementing counter-4 at the sibling of each node on the path *u *→ _*N *_*s*, except at *u*, by *x*.

**counter-6 **If the value of counter-6 is *x *at node *s*, then it is equivalent to incrementing counter-4 at both children (if they exist) of the sibling of each node along the path *u *→ _*N *_*s*, except *u*, and incrementing counter-3 at each node along the path *u *→ _*N *_*s*, except at *u*, by *x*.

In the remainder of this section we first show how to compute the values of these counters, and then the final loss values, at each node in *N*_*u*_.

### Computing the counters

We now describe how the values of the six counters are computed. Initially, each counter at each node in *N*_*u *_is set to 0. Consider any *g *∈ *I*(*G*), and let *g' *and *g" *be its two children. Recall that node *g *must fall under one of the following six categories: 1) *g *is red, 2) *g *is green, 3) *g*, *g'*, and *g" *are all blue, 4) *g *and *g *' are blue, and *g" *is green, 5) *g *and *g' *are blue, and *g" *is red, or, 6) *g *is blue, *g' *is red, and *g" *is green.

Let *a *= ℳ_*N *_(*g*), *b *= ℳ_*N *_(*g'*) and *c *= ℳ_*N *_(*g"*). Also, whenever properly defined, let *a' *= ℳ_Γ, *N *_(*g*), *b' *= ℳ_Γ, *N *_(*g'*) and *c' *= ℳ_Γ, *N *_(*g"*). Based on Lemmas 3 through 8, we now study how the six counters can be updated so as to capture the behavior of losses in each of these cases.

**Case 1**. By Lemma 3, we do nothing in this case.

**Case 2**. Based on Lemma 4, the contribution of any node *g *that satisfies the condition of case 2 can be captured by incrementing the value of counter-1 by one at each node on paths *a *→ _*N *_*b *and *a *→ _*N *_*b*, except at node *a*.

**Case 3**. From Lemma 5 it follows that in this case the contribution of *g *to the loss value changes in a way that is captured by incrementing counter-2 by 1, at each node, except *a'*, on the paths *a' *→ _*N *_*b' *and *a'*→ _*N *_*c'*.

**Case 4**. According to Lemma 6, if *N*_*v *_is regrafted on an edge of *N*_*u *_that is not in *N*_*a'*_, then the contribution of *g *to the loss cost is captured by incrementing counter-3 by 1 at each node except *u *along the path *u *→_*N *_*a'*, and at their siblings. If *N*_*v *_is regrafted on an edge of *N*_*u *_that is in *N*_*a' *_then there are two possible cases:

*a' *≠ *b'*: Recall that *b" *represents the child of *a' *along the path *a' *→ _*N *_*b'*. Here, the contribution of *g *to the loss cost is captured by (i) incrementing counter-3 by two at node *b"*, (ii) incrementing counter-2 by one at each node except *b" *along the path *b" *→_*N *_*b''*, (iii) incrementing counter-3 by one at the sibling of *b"*, and (iv) incrementing counter-1 by one at each node except *a' *on the path *a' *→ _*N *_*c'*.

*a' *= *b'*: In this case, the contribution of *g *to the loss cost is captured by (i) incrementing counter-3 by one at both children of *a'*, and (ii) incrementing counter-1 by one at each node except *a' *on the path *a' *→ _*N *_*c'*.

**Case 5. **By Lemma 7, for this case, the change in the loss contribution of *g *is captured by incrementing counter-5 by 1 at node *a'*, and by incrementing counter-4 by 1 at both children of a' in *N*.

**Case 6. **By Lemma 8, for this case, the change in the loss contribution of *g *is captured by incrementing counter-6 by 1 at node *a'*, and by incrementing counter-4 and counter-2 by 1 each at both children of *a' *in *N*.

#### Computing the final loss values

Our algorithm considers each internal node of gene tree *G*, one at a time, and updates the relevant counters at the relevant nodes in *N*_*u*_, as shown in the previous subsection. Then, based on the counters, it computes, at each node *s *∈ *V *(*N*_*u*_) the value *α *(*s*) = *Loss*(*G*, *S'*) - *Loss*(*G*, *N*), where *S' *= SPR_*N *_(*v*, *s*); this can be achieved by performing a constant number of pre- and post-order traversals of *N*_*u*_. A final traversal of the tree now allows us to compute the value *Loss*(*G*, *S'*) = *a*(*s*) + *Loss*(*G*, *N*) at each *s *∈ *V *(*N*_*u*_). Due to space limitations, a more complete description of the algorithm is omitted from this manuscript (but is available in [29]).

For simplicity in stating the time complexity of our algorithm, we assume that all *G *∈  have approximately the same size. Recall that *n *= | *Le*(*S*)|, *m *= | *Le*(*S*)| + | *Le*(*G*)| and *k *= ||.

**Lemma 9**. *The *SPR-*RS problem on the input instance ⟨*{*G*}, *S*, *v*⟩ *can be solved in O*(*m*) *time*.

*Remark on Lemma 9*: Observe that in cases 2, 3 and 4 (from the previous subsection), handling each *g *might require updating the counters at Θ(*n*) nodes, yielding a total time complexity of *O*(*nm*) for these cases. However, it is still possible to obtain the *O*(*m*) time bound.

Thus, we have the following theorem.

**Theorem 1**. *The *SPR-*RS and *SPR-*S problems can be solved in O*(*km*) *and O*(*kmn*) *time respectively*.

The time complexity of the best known (naïve) solution for the SPR-S problem is Θ (*kmn*^2^). Our algorithm improves on this by a factor of *n*.

### Speeding-up the TBR local search problem

Intuitively, a (rooted) TBR operation may be viewed as being like an SPR operation except that the TBR operation allows the pruned subtree to be arbitrarily rerooted before being regrafted. The TBR-S problem is defined analogously to the SPR-S problem.

Observe that there are Θ (*n*) different ways to select a subtree of *S *to be pruned. Furthermore, there are *O*(*n*) different ways to reroot the pruned subtree. The idea is to directly use the solution to the SPR-RS problem to compute the duplication and loss costs for the *O*(*n*)-cardinality subset of TBR_*S *_defined by any fixed pruned subtree and its fixed rooting. This yields the following theorem.

**Theorem 2. ***The *TBR-*S problem can be solved in O*(*kmn*^2^) *time*.

This improves on the best known solution for the TBR-S problem by a factor of *n*.

### The deep coalescence cost model

Our algorithms for efficient SPR and TBR local searches for the duplication-loss model apply directly to the corresponding SPR and TBR local search problems for the deep coalescence model. This can be achieved using any of the following two methods. The first method is to make use of the result of Zhang [15] who showed showed that if *G *is a uniquely leaf-labeled gene tree and *S *is a species tree such that *Le*(*G*) = *Le*(*S*), then the deep coalescence cost of *G *and *S *is equal to *Loss*(*G*, *S*) - 2| *Dup*(*G*, *S*)|. Thus, our algorithms imply that we can compute the deep coalescence cost of each tree in the SPR (resp. TBR) neighborhood of *S *in *O*(*n*^2^) (resp. *O*(*n*^3^)) time. The second method is to use the algorithm for computing losses, presented in this paper, and slightly modifying it to directly compute the deep coalescence cost. Owing to the similarity in the definition of the loss cost and the deep coalescence cost, this can be done in a straightforward manner (details omitted for brevity). Overall our algorithms yield speed-ups of a factor of *n *over the fastest current approaches for SPR and TBR local searches for the deep coalescence cost model.

## Results

To evaluate the performance of our novel local search algorithms, we implemented our algorithm for the SPR-S problem as part of a standard search heuristic for the duplication-loss problem. We refer to our program as *DupLoss*. We first evaluated its performance on randomly generated gene trees. These data sets represent extreme examples of incongruence among gene trees, and thus, they provide a challenging way to test the run-time performance of a gene tree reconciliation method. The input gene trees for each run consisted of 20 trees, each with the same set of taxa and with random binary topologies and random assignment of leaf labels. We conducted runs with 50, 100, 200, 400, and 1000 taxa in each gene tree. All analyses were performed on a 3 Ghz Intel Pentium 4 CPU based PC with Windows XP operating system. We compared the performance of DupLoss with the program GeneTree [20], which, like Mesquite [21], implements similar local search heuristics based on the best known (naïve) algorithm for the SPR-S problem. Our implementation shows a tremendous improvement in runtime and scalability compared to GeneTree (Table 1). For example, on the 200 taxon data set, our implementation finished in less than five minutes, while GeneTree ran for almost six days (Table 1). Furthermore, we could not run GeneTree when the input trees had more than 200 taxa.

**Table 1:** GeneTree vs. DupLoss. Comparison of the runtimes of GeneTree and DupLoss on the same randomly generated datasets. Times are given in days(d), hours(h), minutes(m), and seconds(s).

Taxa size	GeneTree	DupLoss
50	11 m:42 s	5 s
100	3 h:57 m	33 s
200	5 d:19 h:49 m	4 m:24 s
400	-	43 m:08 s
1000	-	19 h:27 m

We also tested the performance of DupLoss using several empirical data sets. First, we ran our implementation on the 8-taxon, 106-gene yeast data set of Rokas et al. [30], and the 8-taxon, 268-gene Apicomplexan data set of Kuo et al. [31]. For the yeast data set, we made the gene trees using maximum likelihood implemented in RAxML [32], and for the Apicomplexan data set, we used the gene trees included as supplemental data [31]. Tree searches for both data sets finished within one second, and the topologies were consistent with the unrooted species trees presented in each study. Finally, we ran our implementation on a plant data set consisting of 18, 896 gene trees from 136 taxa, previously used in a gene tree reconciliation analysis based on duplications only [33]. Although this is among the largest data sets used in a gene tree reconciliation analysis, our heuristic finished in approximately 24 hours, and the resulting species tree was consistent with the general consensus of plant relationships. DupLoss is freely available upon request.

## Discussion

The novel algorithms presented in this paper enable GTP analyses that incorporate gene duplications and losses as well as deep coalescence on a scale that is impossible with previous implementations. Whereas most phylogenetic analyses only use data from putative orthologs, the duplication-loss model allows one to incorporate data from large gene families into phylogenetic analysis, and it does not depend on the accuracy of orthology estimates. Incorporating deep coalescence events may be critical when there is a history of rapid speciation, and these evolutionary scenarios represent many of the most difficult, and interesting, phylogenetic problems.

Previous advances in GTP heuristics for the duplication cost problem [22,23,34,35], that is, calculating the species tree based on the number of duplications only without considering losses, have enabled promising phylogenetic analyses of extremely large data sets (e.g., 136 taxa and 18, 896 genes in [33]). With partial sequence data, it is difficult, if not impossible, to distinguish losses from missing sequence data. Thus, it has been argued that the duplication only reconciliation cost is more appropriate than the duplication-loss cost when analyzing incomplete data sets (e.g., [36]). However, with the rapid accumulation of complete genome sequences, the duplication-loss problem provides a more complete model of evolution than the duplication problem. Similarly, there has been much recent interest in the deep coalescence problem (e.g., [14]), but it has not been applied to large data sets due to a lack of efficient heuristics.

While GTP has been effective on small data sets (see review in [36]), its performance in general is relatively uncharacterized, largely due to the lack of fast implementations. The parsimony approach minimizes the number of evolutionary events that are counted, and therefore, it may not be appropriate when genes exhibit high rates of duplication and loss or deep coalescence events. In such cases, inferring the species tree based on a likelihood model of gene evolution may be more appropriate. Still, likelihood methods are often computationally burdensome, and the computational difficulties are compounded by the extremely large data sets that are an inherent part of genome-scale analysis. The algorithms in this paper provide a pragmatic solution to the problem of addressing complex processes of evolution on enormous data sets in a phylogenetic analysis.

## Conclusion

The abundance of new genomic sequence data presents new challenges for phylogenetic inference. Genome-scale phylogenetic analyses must account for complex evolutionary processes, such as gene duplication and loss, incomplete lineage sorting (deep coalescence), or horizontal gene transfer, that can produce conflict among gene trees, and they must be computationally feasible for enormous data sets. Our new algorithms for fast local tree search for inferring species trees under the duplication-loss and deep coalescence reconciliation costs expand both the size of the data sets and the range of evolutionary models that can be incorporated into genome-scale phylogenetic analyses.

## Competing interests

The authors declare that they have no competing interests.

## Authors' contributions

MSB was responsible for algorithm design and program implementation, and wrote major parts of the paper. JGB performed the experimental evaluation and the analysis of the results, and contributed to the writing of the manuscript. OE supervised the project and contributed to the writing of the paper. All authors read and approved the final manuscript.
